# Effects of dietary incorporation of *Moringa oleifera* leaf meal on hatching characteristics and serum parameters of local Guinea fowl (*Numida meleagris*) in Togo

**DOI:** 10.5455/javar.2025.l980

**Published:** 2025-12-25

**Authors:** Patrik Nukunu Komi Atitso, Kokou Voemesse, Aduayi Akue, Hèzouwè Tchilabalo Meteyake, Kafui Amivi Tete-Benissan

**Affiliations:** 1 Regional Center of Excellence in Avian Sciences (CERSA), University of Lomé, Lomé, Togo; 2 Togolese Institute of Agronomic Research (ITRA), Lomé, Togo; 3 Forestry Research Laboratory, Faculty of Science, University of Lomé, Lomé, Togo

**Keywords:** Blood parameter, fertile eggs, *Moringa*

## Abstract

**Objective:**

This study evaluated the effects of *Moringa oleifera* leaf meal incorporation in local Guinea fowl breeders’ diet on the hatching rate, quality, and serum parameters of Keet in Togo.

**Materials and Methods:**

For this study, 512 breeder Guinea fowls (128 males and 384 females) were assigned randomly to four dietary groups (with four replicates each), named DT0, DT1, DT2, and DT3, containing, respectively, 0%, 0.5%, 1%, and 1.5% of *M. oleifera* leaf powder in diets. Guinea fowls are raised in confinement. Four incubations (I38, I42, I46, and I50) of 560 eggs were carried out each (Incubated at 37.7°C, with a relative humidity of 55%, the eggs were turned once per hour at a 90° angle until 23 days before transferred to hatching baskets for 3–4 days). These eggs were collected from 384 local Guinea fowls at 38, 42, 46, and 50 weeks of age. Egg weight loss, the duration of hatching events, fertility, hatchability, embryo mortality, and serum parameters of keets at hatch were evaluated.

**Results:**

The duration of the hatching events in the DT0 and DT1 treatments was higher than that of the DT2 and DT3 groups. The fertility rate (%) in DT2 and DT3 (76.43 ± 3.8 and 76.15 ± 4.5, respectively) was higher than that of DT0 and DT1 (73.3 ± 6.6 and 74.2 ± 4.7, respectively). Hatchability (%) was lower in DT0 (81.5 ± 3.3) compared to DT1, DT2, and DT3 (87.54 ± 5.5, 87.03 ± 3.2, and 88.25 ± 4.1, respectively). Embryo mortality rate (%) reduced in DT1, DT2, and DT3 (9.92 ± 0.24; 11.08 ± 0.34; and 11.12 ± 0.71, respectively) compared to DT0 (12.84 ± 1.59). The total proteins and albumin levels were higher in DT1, DT2, and DT3 compared to DT0. Alanine aminotransferase, aspartate aminotransferase, urea, uric acid, creatinine, high-density lipoproteins-cholesterol, and triglycerides levels for all treatments were not significantly different. Total cholesterol decreases in DT1, DT2, and DT3 compared to DT0.

**Conclusion:**

In conclusion, *M. oleifera* leaf meal incorporated into local Guinea fowl breeders’ diets at 0.5%, 1%, and 1.5% improves keets production. However, according to the results on egg production performance of Guinea fowl breeders obtained in our previous study, the best performance was obtained with dietary inclusion of 0.5% and 1% *M. oleifera* leaves powder.

## Introduction

The breeding of local Guinea fowls (*Numida meleagris*) is considered a sector for poverty reduction in West African countries, especially in Togo, and contributes to meeting the population’s animal protein requirements [1,2]. Despite its nutritional and cultural significance [1,3], Guinea fowl breeding still faces many difficulties, such as seasonal reproduction of local Guinea fowl, the low availability of fertile eggs and day-old keets (post-hatch juveniles), and their low growth and high mortality rate during the start [1,3,4]. However, Sodjedo et al. [2] reported that Guinea fowl can lay throughout the year if fed *ad libitum* with adequate feed.

In poultry, several factors, including genetics, breeders’ diet, egg storage duration, and incubation parameters, affect hatchability and the quality of day-old chicks [5–7]. Thus, studies have shown the effects of breeders’ diet on the quality of hatching eggs, hatchability, and the quality of day-old chicks or keets [7-10]. Mustafa et al. [11] showed an improvement in the hatching rate, blood parameters, and immunity of Japanese quails by using feed additives. A positive effect on hatching rate and weight at hatch was also observed by Aberbour et al. [12] using rosemary essential oil in quails.

*Moringa oleifera*, a leguminous plant rich in quality nutrients and secondary metabolites (carotenoids, flavonoids, tannins, saponins, terpenoids, glycosides, phenolic compounds, and so on) which, by different mechanisms of action, give it antioxidant, antimicrobial, anti-inflammatory, hepatoprotective, hypolipidemic, and other properties [8,9,13,14]. It is used for both human and animal consumption due to its nutritional and phytotherapeutic properties [15-17]. In poultry, an improvement in fertility and hatching rate was observed in aged broiler breeders with dietary supplementation of *Moringa* leaf extract [18]. Habibi et al. [19] also demonstrated an increase in hatchability with 1% dietary supplementation of either the powder or hydroalcoholic extract of *M. oleifera* seeds in Chukar partridges. When incorporated up to 15% in the diet of Japanese quail (partially replacing soybean meal), *M. oleifera* had no detrimental effects on egg quality, fertility, and hatchability of quail eggs [20]. Hatching rate and day-old chick weights increased with the injection of 0.5 μg/ml of *M. oleifera* leaf extracts into fertile eggs of Isa Brown breeders [8].

Despite all the earlier reports on *M. oleifera* leaves as a phytogenic feed additive, scientific reports regarding its dietary incorporation by local Guinea fowl breeders on the productive and reproductive performances are scarce. Hence, this study aimed to investigate the effect of incorporating *M. oleifera* leaf powder into the diets of local Guinea fowl breeders on the hatchability of eggs, the performance of keets, and their blood parameters.

## Material and Methods

### Ethical approval

All experimental procedures were conducted in accordance with the guidelines approved by the Animal Ethics Committee of the University of Lomé, Togo (Approval No. 008/2021/BC-BPA/FDS-UL).

### Study areas

The rearing phase of the Guinea fowl breeders that provided the eggs for incubation was studied by the same authors in the same study areas, and the results were published [10]. The University of Lomé in Togo, through the experimental unit of the Regional Center of Excellence in Poultry Science, served as the setting for our study. The Poultry Production Techniques laboratory of the Center provided the Guinea fowl used for the study.

### Moringa oleifera leaves collection and experimental diet formulation

The collection of *M. oleifera* leaves (MOL) for the rearing phase of Guinea fowl breeders, which provided the eggs for incubation, was studied by the same authors, and the results were published [10]. Thus, before their incorporation into feed, the leaves were air-dried on a clean surface. Table 1 presents the phytochemical group concentrations and mineral element composition of *M. oleifera* leaf powder. The *M. oleifera* leaf meal was incorporated into the tested diets at rates of 0%, 0.5%, 1%, and 1.5%. The composition of the different experimental diets (iso-nitrogenous and iso-caloric) is presented in Table 2.

**Table 1.** Phytochemical group concentrations and mineral element composition of *Moringa oleifera* leaf powder.

Phytochemical group	Concentrations
Total phenols (μg Eq Gallic Acid/mg)	125.63 ± 2.07
Tannins (μg Eq Gallic Acid/mg)	87.60 ± 2.65
Flavonoids (μg Eq Rutin/mg)	176.44 ± 10.45
Polysaccharides (μg Eq Glucose/mg)	402.80 ± 12.07
Mineral element	Composition (mg/100g)
Calcium	2455 ± 151
Phosphor	354 ± 22
Magnesium	510 ± 45
Potassium	2062 ± 118
Zinc	2.92 ± 0.6
Copper	2.15 ± 1.2
Manganese	12 ± 12.07
Iron	20 ± 5.7
Selenium	0.32 ± 0.03

Source: (The *Moringa oleifera* leaves used in this work were collected from the same source as reference [59]).

**Table 2.** Composition (%) of experimental diets according to *Moringa oleifera* treatments during 22–31 weeks of age and 32–50 (laying period) weeks of age of Guinea fowl.

Ingredient	Feed composition according to age and group							
	22–31 week of age				32–50 week of age			
	DT0	DT1	DT2	DT3	DT0	DT1	DT2	DT3
Maize	54	53.5	53.5	53	59	59	59	59
Wheat bran	17	17	17	17	13	12.5	12	12
Roasted soybean	19	19	18.5	18.5	18	18	18	17.5
Laying concentrate	2	2	2	2	2	2	2	2
oyster shell	7	7	7	7	7	7	7	7
Methionine	0.5	0.5	0.5	0.5	0.5	0.5	0.5	0.5
Lysine	0.5	0.5	0.5	0.5	0.5	0.5	0.5	0.5
*Moringa oleifera* leaf	0	0.5	1	1.5	0	0.5	1	1.5
Total	100	100	100	100	100	100	100	100
Calculated analysis								
ME (Kcal/Kg)	2787	2791	2795	2799	2849	2860	2870	2873
Crude protein (%)	17.72	17.67	17.45	17.40	17.06	17.13	17.21	17.13
Calcium (%)	2.27	2.26	2.26	2.26	2.27	2.28	2.29	2.30
Phosphorus (%)	0.53	0.52	0.52	0.52	0.49	0.48	0.48	0.48
Methionine (%)	0.78	0.78	0.78	0.78	0.78	0.78	0.78	0.78
Lysine (%)	1.07	1.07	1.05	1.05	1.03	1.03	1.03	1.03
Methionine + Cysteine (%)	1.02	1.02	1.01	1.01	1	1	0.99	0.99

ME: Metabolizable**e**nergy; DT0, DT1, DT2, DT3: Treatments having received, respectively, 0%, 0.5%, 1%, and 1.5% *Moringa oleifera* leavesin the diet. The rearing lasted 28 weeks.

### Experimental design and data collection

For this study, four incubations (I38, I42, I46, and I50) of 560 hatching eggs each were carried out. Each incubation was considered a repetition. These eggs were collected from 384 local Guinea fowl breeders at 38, 42, 46, and 50 weeks of age, divided randomly into four dietary treatments (the Guinea fowl had an average weight of 1176.7 ± 2.9 gm [10]), with four replicates each, 24 females for 8 males [2] per replicate. These are the treatments DT0 (fed with a diet containing 0% MOL meal), DT1 (fed with a diet containing 0.5% MOL meal), DT2 (fed with a diet containing 1% MOL meal), and DT3 (fed with a diet containing 1.5% MOL meal) [10]. After 1 week of acclimatization, the experimental diets were served *ad libitum* to the Guinea fowl starting at 23 weeks of age [10]. The Guinea fowls were reared on litter at a density of 6 per square meter [21] in an open-sided poultry house, partitioned with wood (2.7 m × 2 m) [10]. During the experiment, water was provided *ad libitum*, and natural light was used (with an average temperature of 25°C during the dry season and 27.3°C during the rainy season) [10].

Thus, for each incubation, 140 eggs per treatment group, stored for 3–7 days, were weighed and individually numbered before being placed in the incubator. The eggs were incubated at 37.7°C and 55% relative humidity. During incubation, the eggs were turned at a 90° angle every hour for 23 days in a Petersime^®^ Vision 96 incubator [22]. The eggs were weighed and candled after 23 days of incubation. Those showing signs of live embryos were transferred to hatching baskets [22,23]. The transferred eggs were individually checked every 3 h to identify internal pipping (IP), external pipping (EP), and hatching [22,23]. Eggs in which the embryo’s beak had pierced the inner shell membrane IP were transferred to a new basket (and examined again individually every 3 h) to detect the time at which the shell (above the air cell) cracked EP [22,23]. Cracked eggs were then placed in new baskets to determine the time of hatching [22,23]. At the end of the hatching period, individual incubation, pipping, and hatching times were recorded to determine their average duration [22,23]. Depending on the treatments, unhatched eggs were recorded, broken open, visually examined, and classified as either infertile or containing dead embryos [22,23]. Organ samples (heart, liver, yolk sac) were collected and weighed from 8 keets per treatment (randomly selected) at hatching. The keets were stunned and slaughtered for the samples [10]. For biochemical analyses, venous blood samples (approximately 2 ml) were collected (from 8 Guinea fowl per treatment) in dry tubes [10]. The collected blood was centrifuged (3,000 rpm) to obtain the serum. Albumin, total protein, alanine aminotransferase (ALT), aspartate aminotransferase (AST), urea, uric acid, creatinine, high-density lipoproteins-cholesterol (HDL), total cholesterol, and triglycerides were then measured [10]. The colorimetric method (using a Mindray BS automatic biochemical analyzer, China) was used to determine these blood parameters. The determinations were done in triplicate.

### Calculated parameters

The relative weight loss of the eggs during incubation was determined according to the following formula:

Egg weight loss = 100 × (egg weight at the start of incubation—egg weight on the 23rd day of incubation) / egg weight at setting.

The durations of IP (time of EP—time of IP), EP (hatching time—time of EP), and hatching (hatching time—time of IP) [22] were calculated, as well as the incubation period (time elapsed between the start of incubation and internal pipping) [22].

The number of infertile eggs, dead embryos, and hatched Guinea fowl chicks per treatment was used to calculate the fertility, embryonic mortality, and hatching rates relative to the total number of eggs according to the following formulas:

Fertility = 100 × (Number of fertile eggs / Number of eggs set);

Embryo mortality rate = 100 × (Number of dead embryos / Number of fertile eggs);

Hatching rate = 100 × (Number of Guinea keet hatched / Number of fertile eggs) [21].

The relative weight of the organ was also calculated (Organ weight × 100 / live weight) [9,10].

### Statistical analysis

After the homogeneity and normality tests with Bartlett’s test, data analysis was performed using ANOVA with GraphPad Prism 8.1 software. Comparison of means (expressed as mean ± standard deviation and as a percentage) between the different treatments was performed using Tukey’s test. Differences were considered statistically significant when *p* < 0.05.

## Results

### Incubation outcomes and embryo mortality

Egg weight, egg weight loss after 23 days of incubation, and incubation duration were similar between treatments (*p* > 0.05). Concerning hatching events, IP, EP, and hatching duration were higher in DT0 and DT1 groups compared to DT2 and DT3 (*p* < 0,05), as shown in Table 3. The weight of Guinea fowl keets at hatch and the embryo mortality rate are presented in Table 3. Although the difference was not significant (*p* > 0.05), keet weight was higher in groups DT1, DT2, and DT3 compared to DT0. Concerning the embryo mortality rate (%), it was higher in the DT0 treatment (12.84 ± 1.59) compared to DT1, DT2, and DT3 (9.92 ± 0.24, 11.08 ± 0.34, and 11.12 ± 0.71, respectively) (*p* < 0.05).

**Table 3.** Effect of dietary *Moringa oleifera* leaf meal incorporation in the diets of Guinea fowl on the incubation and hatching traits of the progenies (Mean ± SD).

Parameters	Groups				*p* -value
	DT0	DT1	DT2	DT3	
Egg weight (gm)	39.79 ± 1.65	40.31 ± 0.95	39.82 ± 0.97	39.99 ± 1.07	0.9781
Egg weight loss (%)	11.2 ± 0.31	12.84 ± 0.48	10.92 ± 0.41	11.27 ± 0.32	0.0758
Incubation duration (h)	553.67 ± 1.44	553.33 ± 0.44	554.67 ± 1.11	553.33 ± 0.88	0.9695
Duration of internal pipping (h)	12.5 ± 1^a^	12.5 ± 0.53^a^	10.5 ± 0.7^b^	10.5 ± 0.53^b^	0.0157
Duration of external pipping (h)	22 ± 0.58^a^	21 ± 1.02^a^	18.5 ± 0.88^ab^	17 ± 0,62^b^	0.0145
Duration of hatch (h)	34.5 ± 0.67^a^	33.5 ± 1.18^a^	29 ± 0.67^b^	27.5 ± 0.58^b^	0.0050
Keets weight (gm)	25.35 ± 1.6	25.61 ± 1.46	25.81 ± 0.79	26.59 ± 2.14	0.2896
Embryo mortality (%)	12.84 ± 1.59^a^	9.92 ± 0.24^b^	11.08 ± 0.34^b^	11.12 ± 0.71^b^	0.0065

DT0, DT1, DT2, DT3: Treatments having received, respectively, 0%, 0.5%, 1% and 1.5% *Moringa oleifera* leavesin the diet; Internal pipping: eggs in which the beak of the embryo penetrated the inner shell membrane; External pipping**:** eggs in which the shell over the air chamber is then cracked [22,23;^ a, b^ Within rows, values not sharing the same letters are significantly different (*p* < 0.05).

### Fertility

**Figure 1.**
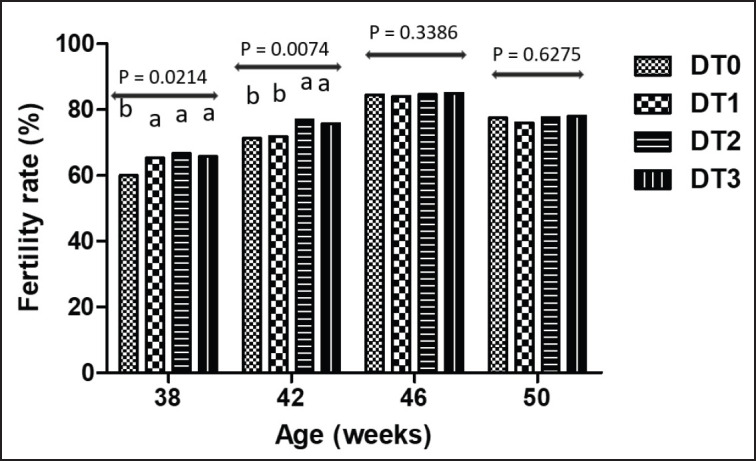
Fertility rate according to guinea fowl breeders’ age (38, 42, 46, and 50 weeks of age) and *Moringa oleifera* treatments. DT0, DT1, DT2, DT3: Treatments having received respectively 0%, 0.5%, 1% and 1.5% *Moringa oleifera* leavesin the diet; ^a, b^ Within columns, histograms not sharing the same letters are significantly different (*p* < 0.05). The rearing lasted 28 weeks.

Fertility rate increased in all treatments until 46 weeks of age before decreasing at 50 weeks. At 38 weeks, fertility was higher in DT1, DT2, and DT3 groups compared to DT0, and at 42 weeks of age, the fertility of treatments DT2 and DT3 was higher than that of treatments DT0 and DT1 (*p* < 0.05). The fertility was similar between treatments at 46 and 50 weeks of age, as shown in Figure 1. On average, fertility increased (*p* < 0,05) in DT2 and DT3 groups (76.43 ± 3.8 and 76.15 ± 4.5, respectively) compared to DT0 and DT1 groups (73.3 ± 6.6 and 74.2 ± 4.7, respectively).

### Hatchability

**Figure 2.**
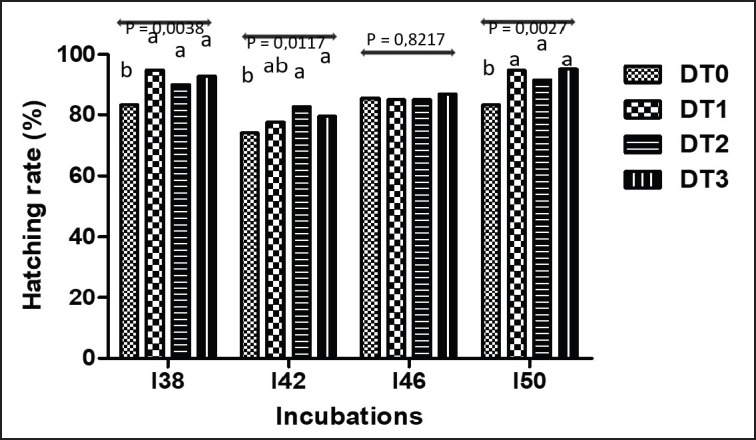
Hatching rate at I38, I42, I46, and I50 incubations according to *Moringa oleifera* treatments. DT0, DT1, DT2, DT3: Treatments having received, respectively, 0%, 0.5%, 1%, and 1.5% *Moringa oleifera* leavesin the diet; I38, I42, I46, I50: incubation of eggs from Guinea fowl breeders aged 38, 42, 46 et 50 weeks, respectively; ^a, b^ Within columns, histograms not sharing the same letters are significantly different (*p* < 0.05).

Figure 2 shows the hatching rate in incubations I38, I42, I46, and I50 according to treatments. Hatchability was significantly higher in group DT2 and DT3 compared to DT0 at I38, I42, and I50 (*p* < 0.05). The difference was not significant between treatments at incubation I46 (*p* > 0.05). On average, treatments DT1, DT2, and DT3 (87.54 ± 5.5, 87.03 ± 3.2, and 88.25 ± 4.1, respectively) recorded a higher hatching rate (*p* < 0.05) than that of treatment DT0 (81.5 ± 3.3).

### Average weight of heart, liver, yolk sac, and keet weight without yolk sac

Heart weight was similar between treatments (*p* > 0.05). Liver weight and keet weight without yolk sac increased in groups DT1, DT2, and DT3 compared to the group DT0 (*p* < 0.05). Yolk sac weight was higher in DT0 treatment compared to DT1, DT2, and DT3 (*p* < 0.05), as shown in Table 4.

**Table 4.** Effect of dietary *Moringa oleifera* leaf meal incorporation in the diets of Guinea fowl breeders on organ weights as well as organ ratio (Mean ± SD).

Parameters		Groups				*p* -value
		DT0	DT1	DT2	DT3	
Average weight (gm)	Heart	0.32 ± 0.04	0.27 ± 0.02	0.27 ± 0.015	0.28 ± 0.01	0.1090
	Liver	0.61 ± 0.01^c^	0.65 ± 0.026^bc^	0.65 ± 0.03^ab^	0.68 ± 0.03^a^	0.0039
	Yolk sac	5.3 ± 0.16^a^	3.43 ± 0.35^b^	3.5 ± 0.46^b^	3.53 ± 0.23^b^	0.0088
	Keet without yolk	18.57 ± 1.14^b^	23.03 ± 0.38^a^	23.83 ± 0.67^a^	23.8 ± 0.83^a^	< 0.0001
Ratio of organ (%)	Heart	1.26 ± 0.03	1.05 ± 0.02	1.05 ± 0.02	1.05 ± 0.017	0.1370
	Liver	2.41 ± 0.01	2.54 ± 0.022	2.52 ± 0.04	2.56 ± 0.035	0.0957
	Yolk sac	20.91 ± 0.18^a^	13.39 ± 0.4^b^	13.56 ± 0.8^b^	13.28 ± 0.4^b^	0.0079
	Keet without yolk	73.25 ± 1.48^b^	89.93 ± 0.48^a^	92.33 ± 0.59^a^	89.53 ± 1.34^a^	< 0.0001

DT0, DT1, DT2, DT3: Treatments having received, respectively, 0%, 0.5%, 1% and 1.5% *Moringa oleifera* leavesin the diet;^ a, b, c^ Within rows, values not sharing the same letters are significantly different (*p* < 0.05).

### Biochemical parameters

Serum parameter concentrations of keets according to treatments are presented in Table 5. Total protein and albumin were significantly higher in DT1, DT2, and DT3 treatments compared to DT0 (*p* < 0.05). Aspartate aminotransferase and alanine aminotransferase of all treatments were not significantly different (*p* > 0.05). The levels of urea, uric acid, creatinine, triglycerides, and HDL-CH were also similar between treatments (*p* > 0.05). Total cholesterol levels were slightly decreased in the DT1, DT2, and DT3 treatments compared to the DT0 treatment (*p* < 0.05).

**Table 5.** Biochemical parameters of Keets at hatch according to *Moringa oleifera* treatments (Mean ± SD).

Parameters	Groups				*p* -value
	DT0	DT1	DT2	DT3	
Total protein (gm/l)	11.3 ± 0.29^c^	32.8 ± 1.1^a^	23.83 ± 0.84^b^	20.5 ± 0.52^b^	< 0.0001
Albumin (gm/l)	5.53 ± 0.33^b^	9.37 ± 1.67^a^	9.67 ± 0.1^a^	7.47 ± 0.1^ab^	0.0271
AST (U/l)	171 ± 8.51	195.67 ± 11.57	188.67 ± 18.24	170.33 ± 5.3	0.1986
ALT (U/l)	7.47 ± 0.93	8.73 ± 0.63	9.07 ± 0.88	7.51 ± 0.34	0.5738
Creatinine (mg/dl)	0.25 ± 0.03	0.37 ± 0.03	0.27 ± 0.03	0.30 ± 0.06	0.2503
Uric Acid (mg/dl)	5.37 ± 0.44	6.35 ± 1.1	5.75 ± 1.6	5.62 ± 0.8	0.9327
Urea (gm/l)	0.25 ± 0.02	0.25 ± 0.01	0.33 ± 0.01	0.28 ± 0.02	0.7184
Triglycerides (gm/l)	1.26 ± 0.13	1.47 ± 0.11	1.18 ± 0.2	1.19 ± 0.06	0.1509
Cholesterol (gm/l)	5.95 ± 0.21^a^	4.25 ± 1^b^	4.63 ± 0.6^b^	4.45 ± 0.23^b^	0.0395
HDL-CH (gm/l)	1.31 ± 0.05	1.37 ± 0.1	1.80 ± 0.12	1.39 ± 0.15	0.2324

DT0, DT1, DT2, DT3: Treatments having received, respectively, 0%, 0.5%, 1% and 1.5% *Moringa oleifera* leavesin the diet; AST: Aspartate Aminotransferase; ALT: Alanine Aminotransferase; HDL-CH: High density lipoproteins-cholesterol;^ a, b, c^ Within rows, values not sharing the same letters are significantly different (*p* < 0.05).

## Discussion

The improvement in fertility with the incorporation of *M. oleifera* leaf meal is comparable to the results of Ghadimi et al. [18], which showed that the extract of *Moringa* leaf in dietary supplementation (200 μl/kg) improves the fertility of aged breeder broilers. Improved fertility has also been observed in New Zealand White male rabbits with the use of an aqueous extract of *M. oleifera* leaves [24], and in Balinese bulls (with supplementation of *M. oleifera* leaves) [25]. Indeed, living organisms, as a result of normal cellular metabolism and sometimes under the influence of environmental factors, produce reactive oxygen species. Part of the antioxidant needed to balance free radicals and avoid oxidative stress is provided by the diet [9,13,16,18]. According to Diemer et al. [26], Asadi [27], Panner et al. [28], Juan et al. [29], Behnamifar et al. [30], and Mohlala et al. [31], oxidative stress has negative impacts on the reproductive functions (protein oxidation in reproductive cells, DNA damage; the reduction in hormonal balance such as FSH (Follicle-Stimulating Hormone) LH (Luteinizing Hormone) which stimulate the production of testosterone, and consequently decreasing circulating testosterone and suppression of sexual behavior), which leads to a reduction in semen quality, egg fertility, and hatchability. Reducing the negative effects of oxidative stress, thereby improving the reproductive function of roosters, can be achieved by supplementing their feed with antioxidants [32]. The antioxidant properties, stimulated by phenolic compounds, carotenoids, flavonoids, flavanols, vitamins C and E, zinc, and selenium, contained in *Moringa* leaves could reduce the negative effects of oxidative stress, thus improving egg fertility [13,16,33,34].

Additionally, *M. oleifera* leaf extract increased serum and testicular testosterone (by inhibiting 6beta-hydroxytestosterone), as well as FSH and LH, semen volume, sperm motility, and sperm viability [24,25,31,35-37]. It increases sexual desire by increasing blood flow to the reproductive organs and stimulating the nervous system [31]. Furthermore, *M. oleifera* reduces the percentage of abnormal sperm and increases testicular weight [38,39]. Additionally, several nutrients, such as selenium and zinc, found in *Moringa* leaves [40], have been shown to improve fertility. Indeed, zinc plays a role in protecting the genetic material in the nucleus of sperm cells, which is essential for successful fertility [41]. Supplementing with plants containing selenium increases fertility and hatching rates [42].

The increase in hatching rate with the incorporation of *Moringa* leaves is consistent with the results obtained by N’nanle et al. [8] in the Isa Brownbreeder’s fertile eggs. This increase in hatchability could be attributed to the availability of energy during the hatching process, which is derived from the metabolism of lipids contained in the yolk, stimulated by *Moringa* leaves [8,43]. This mechanism is confirmed by the present results, which showed a significant reduction in yolk sac weight in treatments DT1, DT2, and DT3 compared to treatment DT0. However, after detachment of the chorioamniotic membrane during pipping, oxygenation and catabolism of lipid are restricted [44,45]. At this point, the glycogen reserves are primarily used by the hatching muscle [44,45]. Likewise, according to De Oliveira [46], *M. oleifera* contains substances promoting the storage of glycogen in the liver. The hydrolysis of this glycogen could produce more energy necessary for muscle contraction [8,47]. These results agree with the significant reduction in IP, EP, and hatching duration observed in groups DT2 and DT3 compared to DT0 and DT1 in this study. The increase in the hatching rate of groups DT1, DT2, and DT3 could also be attributed to several nutrients, such as selenium, zinc, and vitamin E, contained in *Moringa* leaves [14,39] and essential for egg hatching [42,48,49]. Indeed, an increase in the hatching rate with the level of zinc in the diet of laying hens has been reported by Durmus et al. [50]. In addition, the improvement in egg yolk quality because of carotenoids contained in *Moringa* leaves [9,13] could also increase hatching rate. Indeed, Surai and Sparks [51] showed that birds transfer large quantities of carotenoids into the yolk of their eggs. The tissues developing during embryogenesis are, therefore, protected against the damaging effects of free radicals and peroxides by the transferred carotenoids [52].

The similarity in incubation time and egg weight loss could be linked to the similarity in shell ratio of hatching eggs observed in previous work [10]. The similar weight of keets at hatch may have resulted from the high weight of the yolk sac of keets from treatment DT0 compared to DT1, DT2, and DT3; because keet weight without yolk sac of DT1, DT2, and DT3 groups was significantly higher compared to DT0. The high keet weight without yolk sac in DT1, DT2, and DT3 groups could be attributed to the high albumen ratio of eggs from these groups, as shown in our previous study [10], and could be confirmed by the increase in total proteins and albumin of these groups observed in serum parameters results. This improvement in albumen ratio, likely linked to the quality of protein, energy, vitamins, and mineral salts in *Moringa* leaves [10,14], would have, through a positive correlation, improved keet weight without a yolk sac at hatch [5,53].

The reduction in embryonic mortality rate in the MOL incorporated treatments compared to the control treatment can be linked to the reduction in oxidative stress at the end of incubation by the activity of antioxidant enzymes stimulated by flavonoids, polyphenols, flavonols, proanthocyanidins, carotenoids, vitamins C and E, zinc, and selenium contained in *Moringa* leaves [13,34,52].

Concerning serum parameters, the increase in total proteins and albumin in the *M. oleifera* leaves incorporated treatments compared to the control group may be due to the high albumen ratio of their eggs [10], resulting in more protein compounds available for embryo development. The similarity in AST and ALT levels between the different treatments was also reported by Yuangsroi et al. [54] and shows that *M. oleifera* did not have a negative influence on the organs (especially the liver) of Guinea fowl chicks whose parents consumed the leaves. Indeed, the release of transaminases (such as ALT and AST) into the blood indicates liver damage [55,56]. Moreover, the slight reduction in AST and ALT levels (although the difference is not significant) in treatment DT3 compared to DT1 and DT2 could be attributed to the hepatoprotective properties of *M. oleifera* leaves [57]. The reduction in total cholesterol level could be attributed to hypocholesterolemic agents, such as β-sitosterol, contained in *Moringa* leaves [58].

## Conclusion

Results from this study showed that *M. oleifera* leaf meal, incorporated in local Guinea fowl breeders’ diets, improved egg fertility, embryonic viability during incubation, hatchability, and keet quality at hatch. Thus, *M. oleifera* leaves powder, when incorporated into the diets of local Guinea fowl breeders at 0.5%, 1%, and 1.5%, can improve keet production. However, according to the results of our previous study on egg production performance of Guinea fowl breeders, the use of 0.5% and 1% *M. oleifera* leaves in the diet should be encouraged. Further research needs to be done to measure nutrients and bioactive components (amino acids, flavonoids, polyphenols, flavonols, proanthocyanidins, carotenoids, vitamins, and mineral salts, such as zinc, selenium) in local Guinea fowl breeders or their eggs, fed with a diet containing *M. oleifera* leaf powder. Additionally, an investigation is necessary to explore the post-hatch growth performance and blood parameters of keets from Guinea fowl breeders fed a diet containing *M. oleifera* leaves, and to elucidate the mechanisms of action by which *M. oleifera* influences the productive performance of local Guinea fowl.

## List of abbreviations

AST, aspartate aminotransferase; ALT, alanine aminotransferase; CERSA, Regional Center of Excellence in Poultry Science; EP, external pipping; ESTBA, Higher School of Biological and Feed Techniques; HDL-CH: High density lipoproteins-cholesterol; IP, internal pipping; ME, Metabolizableenergy; MOL,* Moringa oleifera* leaves; °C, degree Celsius.

## References

[1] Abdallah N, Oyebamiji OA (2024). Guinea fowl production in Africa: economic importance and constraints. Egypt J Vet Sci.

[2] Sodjedo C, Pitala W, Laré L, Lombo Y (2022). Effect of the season and the sex ratio on the laying and reproductive performance of indigenous guinea fowl (*Numida meleagris*) in South Togo. J Anim Plant Sci.

[3] Abdallah N, Oluwaseun OA (2025). Socio-economic and production dynamics of guinea fowl farming in northern Ghana: insights into health management, challenges, and climate change impacts. Trop Anim Health Prod.

[4] Ousseini MH, Nouri B, Laminou SGM (2024). Production characteristics of local guinea fowl (*Numida meleagris*) in the urban commune of Tessaoua, Niger. Asian J Adv Agricult Res.

[5] Romero-Sanchez H, Enting H, Van Eck L, Van Emous R, Kroetz Neto F, Leentfaar E, et al. (2025). Achieving reproductive performance and quality chicks with modern broiler breeders. J Appl Poult Res.

[6] Gregrova M, Lichovnikova M, Foltyn M, Hampel D (2024). Interaction between broiler parent stock age and egg pre-incubation duration: effects on embryo development, hatchability, day-old chick weight, and yolk sac weight. Animal.

[7] Chang A, Hallez J, Silva MM (2016). Can feeding the broiler breeder improve chick quality and offspring performance?. Anim Prod Sci.

[8] N’nanle O, Té­té-Bénissan A, Tona K, Teteh A, Voemesse K, Decuypere E, et al. (2017). Effect of in ovo inoculation of *Moringa oleifera* leaf extract on hatchability and chicken growth performance. Eur Poult Sci.

[9] N’nanle O, Tété-Bénissan A, Nideou D, Onagbesan OM, Tona K (2020). Use of *Moringa oleifera* leaves in the broiler production chain. 1 – Effect on Sasso breeder hen performance, internal quality of hatching eggs, and serum lipids. Vet Med Sci.

[10] Atitso KNP, N’nanle O, Voemesse K, Lare L, Attivi K, Tete-Benissan AK (2024). Effects of *Moringa oleifera* leaf meal on local guinea fowl breeder hen performance, egg quality, and blood parameters. J World Poult Res.

[11] Mustafa MA, Sabir PS, Mustafa NA (2017). Effect of functional feed additives on egg production, hatchability, and hematological traits of Japanese quails during summer conditions. Iraqi J Agricult Sci.

[12] Aberbour A, Touazi L, Benberkane A, Sofiane A, Sherasiya A, Iguer-Ouada M (2023). Effect of in ovo administration of rosemary essential oil on hatchability, relative hatching weight, and embryo mortality rate in Japanese quail (*Coturnix coturnix japonica*. Animals.

[13] Kashyap P, Kumar S, Riar SC, Jindal N, Baniwal P, Guiné RPF, et al. (2022). Recent advances in drumstick (*Moringa oleifera*) leaf bioactive compounds: composition, health benefits, bio accessibility, and dietary applications. Antioxidants.

[14] Ahmed M, Marrez DA, Abdelmoeen NM, Mahmoud E A, Abdel-shakur Ali M, Decsi K, et al. (2023). Proximate analysis of *Moringa oleifera* leaves and the antimicrobial activities of successive leaf ethanolic and aqueous extracts compared with green chemically synthesized Ag-NPs and crude aqueous extract against some pathogens. Int J Mol Sci.

[15] Pareek A, Pant M, Gupta MM, Kashania P, Ratan Y, Jain V, et al. (2023). *Moringa oleifera*: an updated comprehensive review of its pharmacological activities, formulations, clinical, phytochemical, and toxicological aspects. Int J Mol Sci.

[16] Bibi N, Rahman N, Ali MQ, Ahmad N, Sarwar F (2023). Nutritional value and therapeutic potential of *Moringa oleifera*: a short overview of current research. Nat Prod Res.

[17] Abdel-Wareth AAA, Lohakare J (2021). *Moringa oleifera* leaves as eco-friendly feed additive in diets of Hy-Line Brown hens during the late laying period. Animals.

[18] Ghadimi M, Najafi A, Sharifi SD, Mohammadi-Sangcheshmeh A, Mehr MRA (2024). Effects of dietary *Moringa oleifera* leaf extract on semen characteristics, fertility, and hatchability in aged broiler breeder roosters. Poult Sci.

[19] Habibi H, Kohanmoo MA, Ghahtan N (2021). Effects of different levels of *Moringa oleifera* whole hydroalcoholic extract and seed powder on the hatching rate, nutritional value, and immune response of Chukar partridge eggs. J World Poult Res.

[20] Atuahene CC, Opoku O, Benante V, Quaye B, Adu MA, Tamattey BB, et al. (2020). Effects of *Moringa*
*oleifera* leaf meal on fertility, egg quality, and hatchability of Japanese quails. Ghana J Anim Sci.

[21] Nahashon SN, Adefope NA, Amenyenu A, Wright D (2006). Laying performance of Pearl Gray guinea fowl hens as affected by caging density. Poult Sci.

[22] Kouame YAE, Nideou D, Kouakou K, Tona K (2019). Effect of guinea fowl egg storage duration on embryonic and physiological parameters, and keet juvenile growth. Poult Sci.

[23] Bilalissi A, Meteyake H.T, Oke O.E, Lin H, Onagbesan O, Tona K. et al. (2022). Effect of non-ventilation during the first 10 days of incubation on physiology, hatching events, and post-hatch performance of two commercial layer strains. Eur Poult Sci.

[24] Khalifa WH, Ibrahim FM, El Makawy AI, Sharaf HA, Khalil WKB, Maghraby NA (2016). Safety and fertility-enhancing role of *Moringa oleifera* leaf aqueous extract in New Zealand rabbit bucks. Int J Pharm.

[25] Syarifuddin N, Toleng A, Rahardja D, Ismartoyo I, Yusuf M (2017). Improving libido and sperm quality of Bali bulls by supplementation of *Moringa oleifera* leaves. Media Peternak.

[26] Diemer T, Allen J A, Hales K H, Hales D B (2003). Reactive oxygen disrupts mitochondria in MA-10 tumor Leydig cells and inhibits steroidogenic acute regulatory (StAR) protein and steroidogenesis. Endocrinology.

[27] Asadi N (2017). The impact of oxidative stress on testicular function and the role of antioxidants in improving it: a review. J Clin Diagn Res.

[28] Selvam MKP, Agarwal A, Henkel R, Finelli R, Robert KA, Iovine C, et al. (2020). The effect of oxidative and reductive stress on semen parameters and functions of physiologically normal human spermatozoa. Free Radic Biol Med.

[29] Juan CA, Pérez De La Lastra JM, Plou FJ, Pérez-Lebeña E (2021). The chemistry of reactive oxygen species (ROS) revisited: outlining their role in biological macromolecules (DNA, lipids, and proteins) and induced pathologies. Int J Mol Sci.

[30] Behnamifar A, Rahimi S, Torshizi MAK MAK, Sharafi M, Grimes J (2021). Effects of dietary alpha-lipoic acid supplementation on seminal parameters and fertility potential in aging broiler breeder roosters. Poult Sci.

[31] Mohlala K, Offor U, Monageng E, Takalani NB, Opuwari CS (2023). Overview of the effect of *Moringa oleifera* leaf extract on oxidative stress and male infertility: a review. Appl Sci.

[32] Khalil-Khalili AA, Zhandi M, Zaghari M, Mehrabani-Yeganeh H, Yousefi AR, Tavakoli-Alamooti M (2021). Effect of dietary organic selenium on reproductive performance of broiler breeder roosters under dexamethasone-induced stress. Theriogenology.

[33] Dhalaria R, Verma R, Kumar D, Puri S, Tapwal A, Kumar V, et al. (2020). Bioactive compounds of edible fruits with their anti-aging properties: a comprehensive review to prolong human life.

[34] Carrera-Chávez JM, Jiménez-Aguilar EE, Acosta-Pérez TP, Núñez-Gastélum JA, Quezada-Casasola A, Escárcega-Ávila AM (2020). Effect of *Moringa oleifera* seed extract on antioxidant activity and sperm characteristics in cryopreserved ram semen. J Appl Anim Res.

[35] Wafa WM, El-Nagar HA, Gabr AA, Rezk MM (2017). Impact of dietary *Moringa oleifera* leaf supplementation on semen characteristics, oxidative stress, physiological response, and blood parameters of heat-stressed buffalo bulls. J Anim Poult Prod.

[36] Ajuogu PK, Mgbere OO, Bila DS, McFarlane JR (2018). Hormonal changes, semen quality, and variation in reproductive outcomes of post-pubertal rabbits fed *Moringa*
*oleifera* leaf powder. J Ethnopharmacol.

[37] Momin M, Memiš D (2023). Dietary *Moringa*
*oleifera* leaves to male rainbow trout (*Oncorhynchus mykiss*) broodstock: effects on sperm quality and reproductive performance. Aquaculture.

[38] Zeng B, Luo J, Wang P, Yang L, Chen T, Sun J, et al. (2019). Beneficial effects of *Moringa oleifera* leaf on reproductive performance in mice. Food Sci Nutr.

[39] Ogunlade B, Jeje SO, Adelakun SA, Akingbade GT (2022). *Moringa oleifera* restored semen quality, hormonal profile, and testicular morphology against Highly Active Antiretroviral Therapy-induced toxicity in adult male Wistar rats. JBRA Assist Reprod.

[40] Tarmizi AAA, Ramli NNN, Adam SH, Mutalib MA, Mokhtar MH, Tang S. G. H (2023). Phytofabrication of selenium nanoparticles with *Moringa oleifera* (MO-SeNPs) and exploration of their antioxidant and antidiabetic potential. Molecules.

[41] Tutubalang K, Sebola NA, Mokoboki HK, Mosetle KQ, Manyeula F, Mabelebele M (2022). Inclusion of *Moringa oleifera* leaf meal in the diet of locally bred chickens: effects on growth performance, semen, and hatchability traits. J Appl Anim Res.

[42] Osman AMR, Wahed HM, Ragab MS (2010). Effects of supplementing laying hen diets with organic selenium on egg production, egg quality, fertility, and hatchability. Egypt Poult Sci.

[43] Sulaiman BF (2023). Effect of spraying different concentrations of *Moringa oleifera* leaf extract on broiler breeder hatching eggs. J Kerbala Agric Sci.

[44] Freeman BM (1969). Mobilization of hepatic glycogen in *Gallus domesticus* at the end of incubation. Comp Biochem Physiol.

[45] Pulikanti R, Peebles ED, Keirs RW, Bennett LW, Keralapurath MM, Gerald PD (2010). Pipping muscle and liver metabolic profile changes and relationships in broiler embryos on days 15 and 19 of incubation. Poult Sci.

[46] De Olivera JE (2007). Effect of carbohydrates, hydrolyzed soybean protein, and methionine fed in ovo on turkey poult hatchability and energy status at hatch.

[47] Moran ET (2007). Nutrition of the developing embryo and hatchling. Poult Sci.

[48] Park SY, Kim WK, Birkhold SG, Kubena LF, Nisbet DJ, Ricke SC (2004). Using a feed-grade zinc propionate to achieve molt induction in laying hens and retain post-molt egg production and quality. Biol Trace Elem Res.

[49] Amen HMM, Al-Daraji HJ (2011). Effects of dietary supplementation with different levels of zinc on sperm–egg penetration and fertility traits of broiler breeder chickens. Pak J Nutr.

[50] Durmus I, Ataşoğlu C, Mizrak C, Ertas S, Kaya M (2004). Effect of increasing zinc concentration in the diets of brown parent stock layers on various production and hatchability traits. Arch Anim Breed.

[51] Surai PF, Sparks NH (2001). Comparative evaluation of the effect of two maternal diets on fatty acids, vitamin E, and carotenoids in the chick embryo. Br Poult Sci.

[52] Huang J, Li S, Sung JY, Qiao S, Zeng X, Zhou J (2025). Transfer of antioxidant capacity through placenta and colostrum: β-carotene and superoxide dismutase collaboratively enhance integrated breeding of sows and piglets. Antioxidants.

[53] Iqbal J, Khan SH, Mukhtar N, Ahmad T (2023). Impact of egg size on the quality of eggs, chicks, and post-hatch performance of offspring during the mid-stage of yield (45th week) in Hubbard broiler breeders. Adv Life Sci.

[54] Yuangsroi B, Klahan R, Charoenwattanasak S (2014). Partial replacement of protein in soybean meal by *Moringa oleifera* seed cake in bocourti’s catfish (*Pangasius bocourti*. Songklanakarin J Sci Technol.

[55] Mega A, Marzi L, Kob M, Piccin A, Floreani A (2021). Food and nutrition in the pathogenesis of liver damage. Nutrients.

[56] Abdul H, Ergete W, Tadele A, Woldekidan S, Abebe A, Seyoum G (2023). Toxic effects of 70% ethanol extract of *Moringa* stenopetala leaf (Baker f.) Cufod. (*Moringa*ceae) on the fetus and placenta of pregnant Wistar rats. BMC Complement Med Ther.

[57] Olayemi AT, Olanrewaju MJ, Oloruntoba AC (2016). Toxicological evaluation of *Moringa oleifera* seeds and leaves in Wistar rats. Pharmacogn Commun.

[58] Riry FH, Elkloub K, Moustafa MEL, Mousa MAM, Hanan AH, Youssef SF (2018). Effect of partial replacement of soybean meal by *Moringa oleifera* seed meal on Japanese quail performance during the laying period. Egypt Poult Sci J.

[59] N’nanle O (2018). Utilisation des feuilles de *Moringa*
*oleifera* chez les poules reproductrices et leurs progénitures: Effets sur les paramètres zootechniques et biochimiques.

